# Advances in structure determination by cryo‐EM to unravel membrane‐spanning pore formation

**DOI:** 10.1002/pro.3454

**Published:** 2018-10-18

**Authors:** Harry Scott, Wei Huang, James G. Bann, Derek J. Taylor

**Affiliations:** ^1^ Department of Pharmacology Case Western Reserve University Cleveland Ohio 44106; ^2^ Department of Chemistry Wichita State University Wichita Kansas 67260; ^3^ Department of Biochemistry Case Western Reserve University Cleveland Ohio 44106

**Keywords:** cryo‐EM, membrane attack complex (MAC), cholesterol‐dependent cytolysin (CDC), anthrax, pore structure, membrane

## Abstract

The beta pore‐forming proteins (β‐PFPs) are a large class of polypeptides that are produced by all Kingdoms of life to contribute to their species' own survival. Pore assembly is a sophisticated multi‐step process that includes receptor/membrane recognition and oligomerization events, and is ensued by large‐scale structural rearrangements, which facilitate maturation of a prepore into a functional membrane spanning pore. A full understanding of pore formation, assembly, and maturation has traditionally been hindered by a lack of structural data; particularly for assemblies representing differing conformations of functional pores. However, recent advancements in cryo‐electron microscopy (cryo‐EM) techniques have provided the opportunity to delineate the structures of such flexible complexes, and in different states, to near‐atomic resolution. In this review, we place a particular emphasis on the use of cryo‐EM to uncover the mechanistic details including architecture, activation, and maturation for some of the prominent members of this family.

## Introduction

Through the evolutionary race between hosts and microbial pathogens, a similarly sophisticated strategy has been developed both for attack by foreign invaders and for defense by the targeted host.^1^ Several microbes use pore‐forming protein (PFP)s to disrupt the protective plasma membrane barrier of the infected host.^2^ These proteins are secreted as soluble monomer precursors that are recruited to the plasma membrane of target cells by specifically recognizing either lipids^3^, ^4^, ^5^ or cell surface receptors.^6^, ^7^, ^8^ After an activating event which can include the protease cleavage of the precursor proteins,^9^, ^10^ most PFPs oligomerize to form a prepore structure and subsequently undergo conformational changes to form a mature, membrane‐spanning pore that is characterized by an amphipathic, circular ring‐like structure. PFPs can be classified into α (using α helices to form pores) or β (which form transmembrane β barrels) sub‐types.^11^ In this review, we discuss a family of the β‐barrel pore forming protein (β‐PFP) oligomeric complexes. We place special emphasis on the use of cryo‐electron microscopy (cryo‐EM) as a structural tool and how it has been used to expand on the number of unique PFP structures, which has led to significant advances in our understanding of pore assembly and the maturation processes.

Cryo‐EM is a rapidly growing and powerful tool that was recognized as the “method of the year” in 2015^12^ and its application and associated development in biology warranted the award of the 2017 Nobel Prize in Chemistry.^13^ Cryo‐EM provides structural information by capturing the projections of individual biological molecules embedded in vitrified ice under physiological conditions.^14^, ^15^ Experimental barriers, such as radiation damage, beam‐induced motion, and poor signal‐to‐noise ratio with limited electron dose, have been overcome with improvements in electron beam coherence, better vacuums, increased stability of the sample stage, and perhaps most notably, the introduction of direct electron detectors. Improved detective quantum efficiency of these detectors achieves higher signal‐to‐noise ratios, and fast frame rates enable recording movies to fractionate the dose allowing for the correction of particle motion during imaging.^16^, ^17^, ^18^ These technological and methodological improvements have not only revolutionized the way we understand pore‐forming toxin systems, but also facilitate our ability to see molecular complexes of all types at near‐atomic resolution.

The structures of monomeric units of β‐PFPs have largely been elucidated with the use of X‐ray crystallography. Despite the relatively small mass of β‐PFP monomers (less than 100 kDa), the pore structures are generally composed of multiple copies (from 7 to 50 units^19^ of the protein thereby creating a wide spectrum of pore sizes. Functionally, the pores puncture the cell membrane to disrupt electrolyte balance and/or to translocate foreign peptide chains or intact proteins^20^ into the cytosol of the infected host. The large oligomeric membrane‐spanning pores have long been suitable subjects for negative stain electron microscopy,^21^, ^22^, ^23^ which is limited in terms of resolution that can be achieved. The application of cryo‐EM has provided the ability to bridge the gap between the atomic resolution obtained from the crystal structure of a monomer and the protein‐protein interactions that facilitate pore assembly.^24^ Due to superior resolutions obtained with cryo‐EM (11–2.9 Å resolution, see Supporting Information Table S1),^25^, ^26^, ^27^, ^28^, ^29^ researchers can now analyze these functionally dynamic structures at superior detail. In doing so, new conformational states have been identified that were previously inaccessible by other structural methods.

A number of pathogens have evolved these specialized β‐PFPs as a means of gaining access to the inside of the host cell. Membrane‐spanning pore formation occurs directly on the plasma membrane or is triggered by a pH change that is encountered within endocytic vesicles following endocytosis of the surface‐bound assembly. The β‐PFPs consist of ∼25% of all bacterial cytotoxic proteins.^30^, ^31^ The bacteria that cause pneumonia, meningitis, and septicemia produce and release a cholesterol‐dependent cytolysin (CDC) toxin that binds to the host membrane and assembles into ring‐shaped pore structures that are large enough to allow for the passage of amino acids, nucleotides, proteins, and ions.^32^, ^33^, ^34^ The deadly *Bacillus anthracis* bacterium produces a tripartite toxin system that attacks the host to cause anthrax disease, which is associated with a cascade of events following infection that includes immune system evasion.^35^, ^36^, ^37^ However, the use of PFPs is not restricted to pathogens finding efficient and effective methods of killing the host. In the world of fungi, plants, mammals, protozoa, as well as bacteria, membrane attack complex/perforin‐like family (MACPF) proteins are used as pore‐structures that provide defense mechanisms against their pathogenic counterparts.^38^, ^39^, ^40^, ^41^ As an example, the earthworm *Eisenia fetida* produces a lysenin pore in its coelomic fluid as part of its defense mechanism.^42^ Despite the disparate functions of pathogen‐offensive versus host‐defensive pores, the structures and mechanisms of pore activity are reasonably well conserved across species.^11^

**Figure 1:**
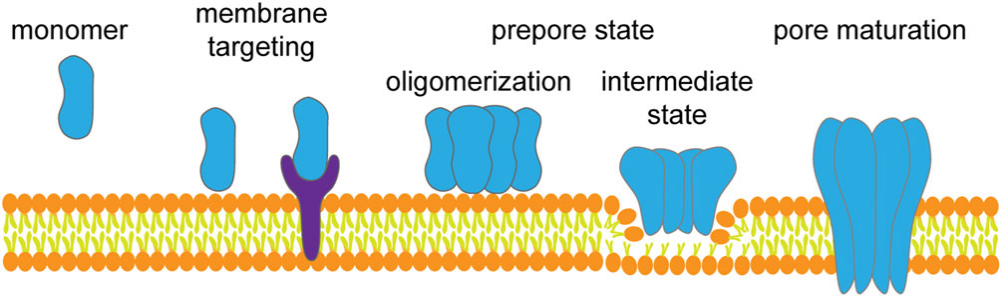
A general scheme for the assembly of pore forming proteins. The monomeric proteins localize on the membrane either by recognition of specific lipids or by receptor binding. A signaling event, such as proteolytic cleavage, triggers the membrane‐associated monomers to oligomerize into a prepore. The prepore matures into a lipid‐spanning pore that includes structural rearrangement to facilitate lipid bilayer penetration.

A few common themes exist in β‐PFP assembly and function (Fig. 1). First, these β‐PFPs must have some way of recognizing the appropriate host cells to target. This recognition is generally accomplished with a specificity motif on the monomeric subunit of the protein or on the pore assembly. Some β‐PFPs recognize the cell membrane itself whereas others recognize a receptor or a class of receptors. Second, most of these β‐PFPs require an activation step which commonly involves proteolysis of the monomeric subunit of the β‐PFPs to initiate oligomerization of the monomeric units to form the pore or an intermediate, prepore structure. Third, after oligomerization and a subsequent triggering event, the β‐PFPs form a β‐barrel pore which results in a perforation of the membrane. This region of the protein is signified in the monomer domain organization as the pore‐forming module (PFM). Lastly, some of these β‐PFP systems deliver specific enzymatic moieties into the cytosol of the cell to intervene in specific signaling pathways.

## Members of β‐Pore Forming Proteins

It is fascinating that mammalian cells have evolved complex killing machinery to fight against bacteria, while bacteria have evolved a highly similar system used to attack its host. While the overall functional structures of the pores are similar, each member of β‐PFPs discussed in this review represent unique domain architectures and distinct modes of action. In this section, we discuss three major families, including MACPF/CDC superfamily, aerolysin family and AB toxin family.

### MACPF/CDC superfamily

The CDCs comprise a superfamily of architectures that include aegerolysin, anemone cytotoxin, fungal fruit body lectin (FB lectin), MACPF, thermostable direct hemolysin (TDH), thaumatin and thiol‐activated cytolysins (see https://pfam.xfam.org/clan/CDC). Membrane penetration and ultimate cell killing proceeds through a multi‐step process that includes selective target recognition events, membrane disruption, dysregulation of the intracellular environment, and propagation in the host cell cytosol.^43^, ^44^, ^45^ Certain gram‐positive bacteria secrete CDC toxins that target the host through the selective recognition of cholesterol in the mammalian cell membrane.^46^ Meanwhile, MACPFs secreted by immune cells implement a series of steps for membrane recognition that begins with activation of the complement system, which is triggered as part of the innate immune response against bacterial pathogens.^47^, ^48^, ^49^ Ultimately, activation of complement leads to the formation of MACPF on the bacterial surface and the formation of the MACPF pore.^50^

The X‐ray crystal structures of individual monomers have been instrumental in understanding and modeling the EM structures of oligomeric pores. For example, the X‐ray crystal structure of the pneumolysin monomeric protein has been fit into the EM map to assemble models of the oligomeric prepore and pore states.^24^, ^51^ In a similar fashion, the X‐ray crystal structure of another CDC toxin, perfringolysin O (PFO),^52^ has been used to model the pore states for many other CDC members. A comprehensive analysis of the CDC superfamily members has revealed that the monomeric proteins are arranged in four distinct domains. Domain 1 (cap domain) sits at the top of the protein, bridging interactions between domain 2 (linker domain) and domain 3 (PFM) which contains 2 alpha helical regions that form transmembrane β hairpin moieties (TMH1 and TMH2) in the pore conformation, and domain 4 (receptor binding domain) is contiguous with domain 2 and forms the initial membrane sequestering region (see Fig. 1, Rosado et al., and the excellent structural analysis of MACPF and PFO therein).^53^ However, unlike other β‐PFP systems, domain 1 of CDCs is not cleaved by a protease, but remains intact.^53^ Domain 2 interacts with TMH1 and the disruption of this interaction is necessary for TMHI to penetrate the membrane.^53^ Once the monomers are assembled into the prepore state, maturation to the pore involves a major conformational change in which domain 2 essentially flattens out, thereby losing its interactions with domain 3. Domain 3 is allowed to unfurl its alpha‐helical TMH1 and TMH2 regions which convert to β‐hairpins that insert into the membrane to form a membrane‐spanning pore.^24^

As the structures of MACPFs/CDCs are highly homologous,^53^, ^54^ the mechanism of pore assembly and formation for these proteins is likely to be conserved as well. The crystal structure of the pneumolysin pore suggests that early association of the monomers on the membrane surface occurs through a side‐by‐side interaction that is weak and includes domains 1 and 4 of the adjacent monomers.^55^ While the mechanism of pore formation is not very clear, the contacts formed between monomers in the prepore state are not predicted to change substantially in the transition to its pore state.^56^ One specific question regarding maturation is whether it is an all‐or‐nothing transition, occurring once the ring‐shaped oligomeric prepore has formed, or if it is a stochastic, noncooperative transition from either the ring‐like prepore state or from arc structures, which are reminiscent of incomplete oligomeric assemblies.^25^ It had also been unclear whether these arc‐like structures represented functional membrane‐spanning pores or whether they were assembly intermediates that led to the eventual formation of circular pores. Only with sub‐tomogram cryo‐EM analysis of pneumolysin^57^ and atomic force microscopy studies of suilysin^25^ was it revealed that both the prepore and the pore conformations included arc‐like structures. Through cryo‐EM, the direct visualization of these structures helped to confirm the existence and precise quaternary organization of these unique architectures.

In the MACPF, activation of the complement pathway ultimately leads to the association of the complement proteins C5b, C6 through C8, and finally C9. Recent work by Serna et al have use cryo‐EM to elucidate the complete, membrane‐bound structure of human MAC.^55^ The 8.5 Å structure shows that the MAC is not assembled into a symmetric, completely closed ring. Rather, the asymmetry of the structure suggests a mechanism of assembly of the pore that transcends directly from its monomeric state. Based on these findings, the authors hypothesize that the mechanism of bacterial killing by PFPs may not simply (and exclusively) be to poke holes in the membrane leading to internal leakage, but that it also involves altering the membrane curvature to disrupt intracellular signaling.

Pore formation and membrane penetration of MACPF is orchestrated by the C9 protein, which shares significant homology with perforin (∼25%) and represents another example of how cryo‐EM has led to significant advancements in understanding the functions of this particular system. C9 can form a contiguous homo‐oligomeric structure, as revealed by the 8 Å cryo‐EM structure of poly C9.^58^ Interestingly, the authors show that the thrombospondin (TSP) domains of C9 pack tightly against each other with the interface situated well above the membrane plane. This organization suggests that C9 proteins that are already associated with the membrane can recruit other C9 proteins through the interactions with the TSP domain. Originally, the lower resolution EM structures did not provide sufficient detail to confidently orient the monomers of perforin correctly within the pore.^59^ It was only after, when the improved resolution of the 8 Å poly‐C9 pore became available, that it was realized that poly C and perforin adopt the same monomer orientation within the pore structures.^58^

### Aerolysin family

**Figure 2:**
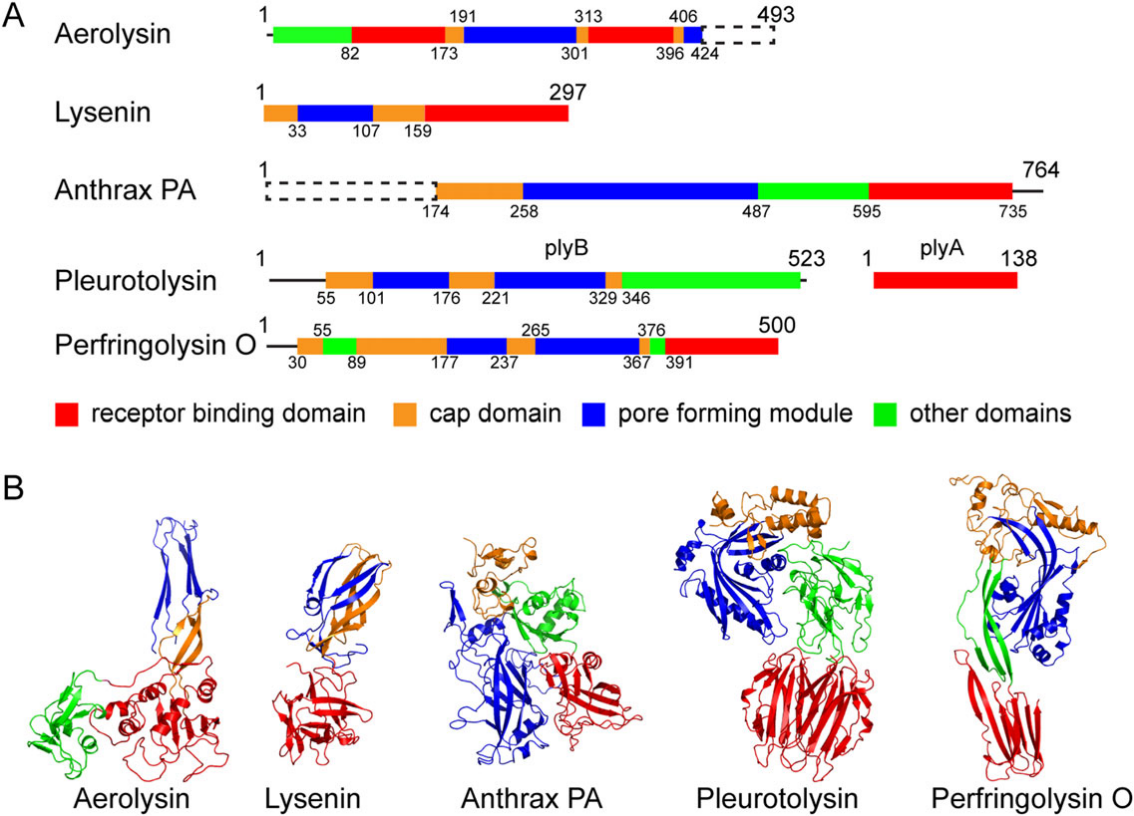
Structures of pore forming proteins. (A) Domain organizations of the pore forming proteins aerolysin, lysenin, anthrax toxin protective antigen, pleurotolysin, and perfringolysin O. The cartoon diagram shows the individual domains color‐coded as indicated. The protease cleaved domain is shown as a dashed rectangle box and the segment without structural information is shown as a solid line. (B) Structural representation of PFP monomers as it exists in the prepore state. The color coding is the same as in panel (A)

The membrane‐spanning pore state structure of lysenin, a member of the aerolysin family, is another example of a β‐PFP that has been solved using cryo‐EM (Supporting Information Table S1). The lysenin monomer is a 33 kDa PFP found in the coelomic fluid of the earthworm *Eisenia foetido.* Lysenin is a component of the earthworm's immune system, exerting its function by attacking membranes of parasites.^42^, ^60^ In addition to the X‐ray structure of the lysenin monomer,^61^ the 3.1 Å structures of the pore, determined independently using cryo‐EM^28^ and X‐ray crystallography,^62^ have helped to identify the functional folds and interactions of the three separate domains of the lysenin monomer [Fig. 2(A,B)]. This information has been critical in depicting the distinct conformational changes of the cap domain, the PFM, and the receptor‐binding domain of lysenin that occur during pore maturation.

**Figure 3:**
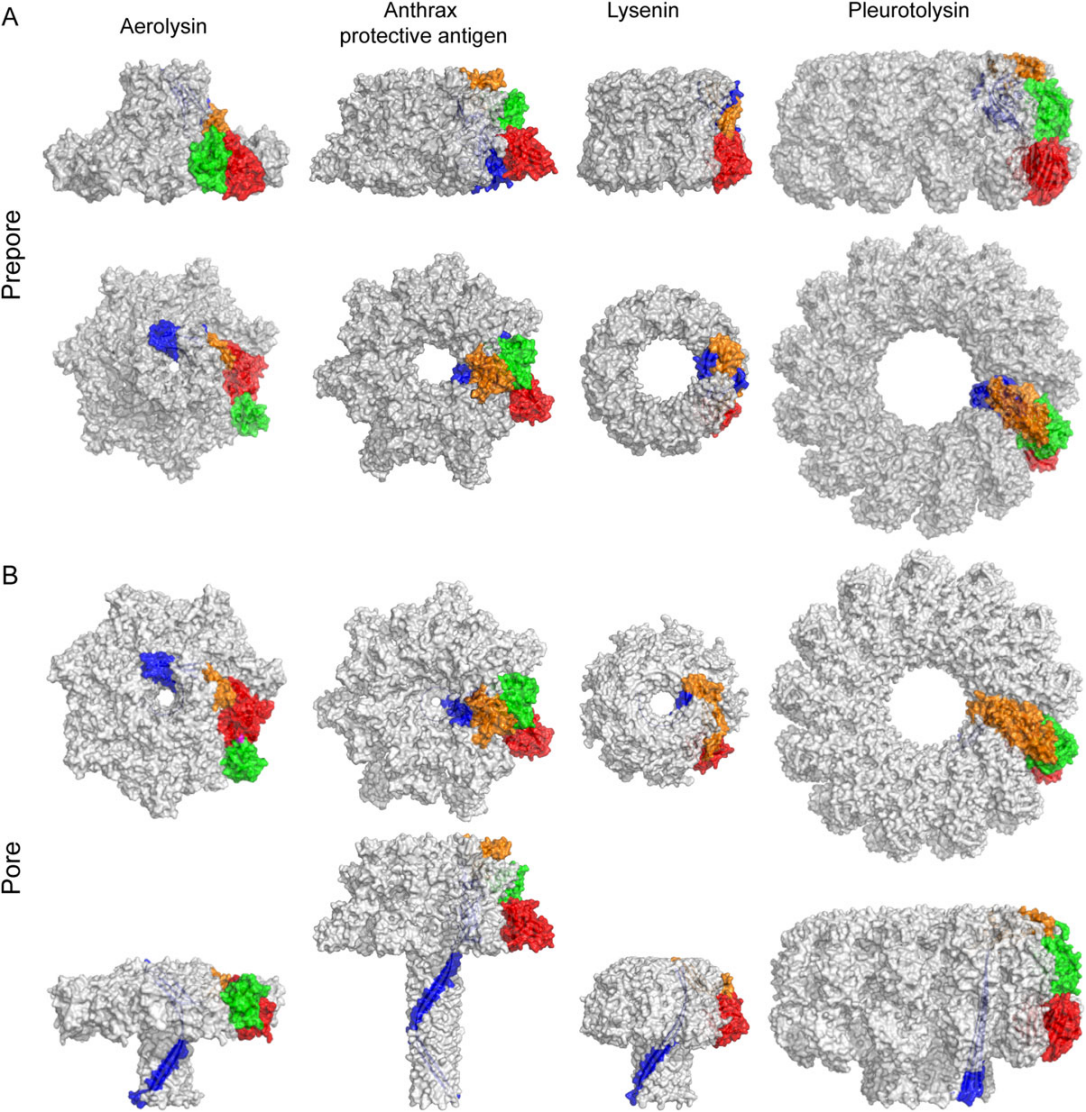
Structural homology among PFPs. The known structures for several PFPs are shown in the prepore (A) and the pore (B) states. The oligomers in both states are shown using surface representation in grey with one monomer color‐coded based on the domain organization described in Figure 1. In each panel, the top view of the oligomer is shown in the top row, with a side view shown in the bottom row

A hybrid approach using the available structural information of the lysenin monomer and the nonameric pore structure provided sufficient information to assemble a model of the nonameric lysenin prepore [Fig. 3(A,B)]. A comparison of the two structural states indicates that the transition from prepore‐to‐pore does not result in any discernable conformational changes of the receptor‐binding domain. In contrast, the cap‐domain exhibits a moderate structural deformation while the PFM undergoes a major conformational alteration, with some residues moving as far as 100 Å, during prepore‐to‐pore formation. The crystal structure of lysenin in complex with sphingomyelin^61^ combined with a bending deformation of the cap domain^28^ further suggests that structural changes triggered in the cap domain might include the molecular sensor that initiates the prepore‐to‐pore transition. These structural data have been instrumental in designing experiments to better understand lysenin pore formation versus substrate interaction events.^61^, ^63^ For example, it was discovered that mutation of Trp20, a residue involved in interchain contacts in the pore structure was unable to oligomerize and became lytically inactive when it was mutated to an alanine residue.^63^, ^64^ In contrast, a double mutation of neighboring residues (Tyr24Ala & Tyr26Ala) abrogated sphingomyelin binding events and also interfered with lytic activity.^61^ This is yet another example that demonstrates the advantage of having multiple structural models, including those obtained by cryo‐EM, to guide the design of separation‐of‐function experiments and to better interpret biological data.

### AB toxin family

A more intricate pore‐forming toxin system belongs to the *Bacillus anthracis* bacterium, which is the causative agent of anthrax. Upon infection, the bacterium secretes the anthrax toxin, a tripartite set of proteins that targets the host cells harboring one or both of its target receptors.^7^, ^65^ There are three complimentary components that are secreted from the bacterium which must interact to form the complete toxin system; the 83 kDa protective antigen (PA_83_) monomer that binds host cell receptors, the MAPKK signal altering lethal factor (LF), and edema factor (EF) which acts as a calmodulin‐dependent adenylate cyclase.^65^, ^66^, ^67^ PA_83_ is comprised of four domains: domain 1 contains a furin cleavage site that regulates oligomeric prepore formation, domain 2 comprises residues that form the β‐barrel of the pore, domain 3 is responsible for oligomerization and domain 4 is responsible for receptor binding. Ultimately, the anthrax monomers oligomerize to form heptameric prepores which are subsequently loaded with LF and/or EF.^68^, ^69^, ^70^ After being endocytosed, the pH‐induced maturation of the prepore to the pore conformation creates a channel from the endosome lumen to the cytosol of the cell. The maturation and complete membrane spanning of the anthrax pore results in lysis of the late endosome or provides a channel through which peptide toxins are translocated into the cytosol to exert their deleterious effects on the infected host.^71^, ^72^, ^73^, ^74^, ^75^, ^76^, ^77^ While the anthrax prepore was solved over a decade ago using X‐ray crystallography,^78^ similar strategies to obtain a high‐resolution structure of the mature pore had remained elusive. Recently, however, the anthrax mature pore was solved to a resolution of 2.9 Å using cryo‐EM and single‐particle reconstruction techniques.^27^ The resulting structure revealed that the pore adopts a “flower on a stem” conformation, which is entirely unique when compared to the structure of the prepore. In the pore configuration, the stem is represented as a transmembrane β‐barrel that is 105 Å in length and 27 Å in width.^27^ In the transition to pore, domain 2 of each individual subunit undergoes a dramatic rearrangement to form β‐hairpins that interact intimately with those of neighboring subunits to form the stem of the pore that is segregated into four unique regions called the mouth, phi‐clamp, throat, and tube.^27^ The mouth is the widest region of the channel, measuring ∼30 Å. The channel constricts appreciably as it approaches the narrowest region of the channel, the phi‐clamp, with a diameter of ∼6 Å.^76^ The next region of the channel, the throat, is ∼16–20 Å in width, and the last region, the tube, measures ∼14–18 Å in width.

Because of such dramatic differences in the structures, the mechanism of anthrax PA_63_ prepore‐to‐pore conversion is not well understood.^79^ However, the monomeric subunit has been solved by X‐ray crystallography at a variety of pH conditions^80^ and the structures, combined with the cryo‐EM data of the assembled pore, provide specific clues to shed light on the mechanism of pore formation. One example is the 2β_10_‐2β_11_ loop (the loop connecting β_10_ and β_11_ strands of domain 2), which exhibits different conformations in separate structures of the prepore assembled as either a heptamer (PDB entry: 1TZO) or as an octamer (PDB entry: 3KWV). These differences highlight a degree of flexibility in this region that is likely to have functional implications. With this in mind, it is worth noting that mutating pH‐sensitive residues (*e.g.* Asp425) within this loop impedes pore‐formation in a dominant‐negative fashion.^81^, ^82^ As such, it is possible that the loop is directly responsible for sensing changes in pH leading to conformational alterations that relay additional rearrangements of adjacent loops. Ultimately, the combined changes could disrupt hydrogen‐bonding networks and allow for coordinated structural rearrangements resulting in the membrane insertion loop forming the β‐barrel in the pore state.^27^

## Modules of β‐Pore Forming Proteins

**Figure 4:**
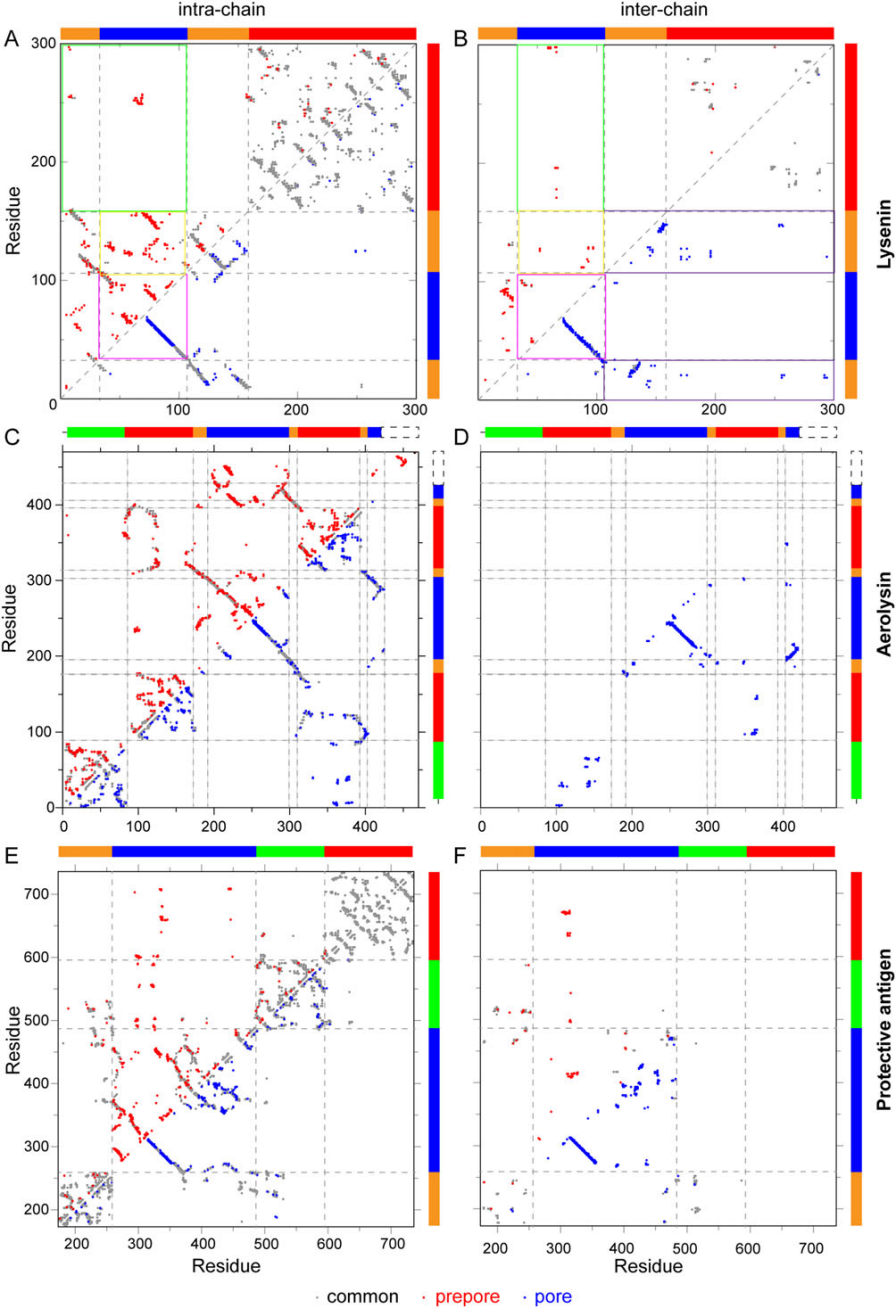
Contact map analysis of the prepore and the pore states. Intrachain (left column) and interchain (right column) contacts observed in the prepore and the pore states of lysenin (A‐B), aerolysin (C‐D) and protective antigen (E‐F). A contact was defined as atom‐atom interactions that reside between two non‐neighboring residues as calculated by the program “Contacts of Structural Units.”^83^ Common contacts observed in both states are shown in grey, unique contacts in the prepore and the pore states are shown in red and blue, respectively. Interchain contacts include, and are exclusive to, the two neighboring subunits that are immediately adjacent to the individual subunit being analyzed. The nanomeric prepore structure of lysenin was assembled based on the model of the pore structure

β‐PFPs are multi‐domain proteins, exerting their functions through a series of step‐wise conformational states. The different interactions formed within the pore and prepore state can be described in great detail using contact map analyses plots (Fig. 4). Such analyses help to identify the multiple interactions that are broken and reformed during maturation for Lysenin, Aerolysin, and PA complexes (Table 1). The consensus feature is that each β‐PFP needs to disrupt intrachain contacts in the prepore states to form new interchain contacts in the pore state, while the total number of contacts remains about the same. The increased interchain contacts in the pore state is likely necessary to maintain an enhanced stability of the pore as compared to the prepore.

**Table 1:** Total Number of, and Changes in, Molecular Contacts for Prepore and Pore States of Three β‐PFPs as Determined from Contact Plot Analyses

	State	# of intrachain contacts	# of interchain contacts	# of total unique contacts	# of total common contacts (intra/inter)
Lysenin	Prepore	319	126	445	555/49
	Pore	188	238	426	
Aerolysin	Prepore	838		838	652/0
	Pore	492	270	762	
PA	Prepore	446	77	523	1457/97
	Pore	266	254	520	

### Pore forming module

Of the individual domains, the PFM undergoes the largest conformational rearrangement when β‐PFPs transition from the prepore to the pore state. A thorough description of contact map interpretations, using PA as an example, highlights the multiple molecular interactions that are lost and formed during pore maturation (Supporting Information Fig. S1). The majority of the unique intradomain contacts of the globular domain 2 in the prepore state [Supporting Information Fig. S1(A); red dots in Supporting Information Fig. S1(B)] are altered significantly as the PFM of domain 2 gets extended and forms new contacts in the mature pore [Supporting Information Fig. S1(C); blue dots in Supporting Information Fig. S1(B)]. Additionally, the intradomain contacts formed between domain 2 and domains 3 and 4 [Supporting Information Fig. S1(B) yellow dash box] in the prepore state are disrupted to enable the release of the compact form of PFM so that it can become extended in the pore state (Supporting Information Fig. S1).

The PFM modules of β‐PFPs can be classified based on secondary structure rearrangements in the membrane insertion region. For instance, in Class I PFMs that include lysenin and aerolysin, the membrane insertion regions adopt a β‐strand conformation in the prepore state. Conversely, Class II PFMs, such as pleurotolysin, anthrax protective antigen (PA) and perfringolysin O, have membrane insertion regions that are primarily helical in structure or which lacks a defined structure in the prepore state [Fig. 2(B)]. In the case of lysenin the cap domain forms a linear rod shape with the PFM wrapped around it on one side [Figs. 3(A) and 4(A), yellow box], and another PFM of the neighboring chain packing with it on the opposite side [Fig. 4(B), yellow box]. Part of the PFM is sandwiched between the cap domain and the receptor‐binding domain [Fig. 4(A), green box], which also contacts the neighboring receptor‐binding domain [Fig. 4(B), green box]. During the formation of the lysenin pore, the bending deformation of the cap domain [Fig. 3(B)] occludes the PFM, making contacts with both the neighboring receptor‐binding domain and the neighboring cap domain [Fig. 4(B), purple boxes]. These mutually exclusive contacts stabilize their corresponding states (prepore or pore), so that the switch to the other set of contacts most likely contributes to the molecular driving force that propagates maturation. Consequently, disruptions of native contacts of the PFMs in the prepore state (Fig. 4, red dots in magenta boxes) enables the formation of new intrachain and interchain contacts (Fig. 4, blue dots in magenta boxes) that facilitate the formation of the β‐barrel pore. Morphing analyses between several prepore and pore states suggests that β‐PFP maturation involves a swirling membrane insertion mechanism of the PFM.^28^, ^84^

Regardless of the sequence variations among PFMs, the structural topologies are highly conserved in Class I PFMs.^61^, ^85^ The Class I PFMs do not need to undergo a secondary structure rearrangement during the transition from prepore‐to‐pore. Class II PFMs seem to require a more intricate mechanism that includes the breaking of bonds that stabilize the α‐helix so that it can transition into β‐strands. Amyloid beta‐peptides (Aβ), which form misfolded oligomers during the development of Alzheimer's disease, possess a helical structure in the N‐terminal region and its transition to a β‐strand conformation coincides with aggregation of peptides.^86^, ^87^, ^88^ In the case of anthrax PA, a loop residing between the β_3_ and β_4_ strands of domain 2 (2β_3_‐2β_4_) contains a helical region that acts in a similar manner as the amyloid Aβ peptides. In this scenario, however, exposure to low pH seems to drive the α to β switch of the 2β_3_‐2β_4_ loop during PA pore maturation. The molecular contacts of domain 2 in the prepore and pore states also differ significantly within and between individual monomers of the assembled PA heptamer [Fig. 4(E,F)]. Taken together, these data suggest a mechanism of maturation that includes a pH sensor that initiates maturation^27^, ^80^, ^89^ and is followed by changes in molecular contacts that are necessary to completely drive PA conversion from prepore‐to‐pore.

As an alternative to the previously mentioned classifications, PFMs can also be typified based on the components of the membrane insertion region. For type I, the membrane insertion regions of PFMs are characterized by two β‐strands connected by a loop. As such, type I PFMs include lysenin, anthrax PA, aerolysin and monalysin. By comparison, type II PFMs include pleurotolysin, perfringolysin O and suilysin, and are categorized by the existence of two membrane inserting loops residing in different sequence segments, which connect a pair of β‐strands [Fig. 2(A)]. The type of membrane insertion loop appears to be a determinant for the pore size as type I PFMs tend to form small size pores that allow the passage of ions or polypeptide chains, while type II PFMs generally form a larger pore size that enables the permeation of folded proteins [Fig. 3(B)].

### Protease cleaved domain

Some β‐PFPs contain a protease‐cleaved domain that plays an integral role in the assembly of the prepore or maturation of the pore. In some cases, the formation of a functional pore proceeds through the cleavage of high molecular weight biosynthetic precursors. For example, aerolysin is first released from the cell as a protoxin that is less susceptible to aggregation and activated upon the proteolytic cleavage of a C‐terminal peptide in proaerolysin.^90^, ^91^ Structural analysis of proaerolysin by X‐ray crystallography^84^, ^92^ and the aerolysin pore structure solved by cryo‐EM^29^ reveals that this protease cleaved domain [segment with dashed box in Fig. 4(C)] interacts with the PFM [segment in blue in Fig. 4(C)] through a network of intrachain contacts. These contact sites on the PFM are exposed upon the release of the protease cleaved domain, enabling the establishment of interchain contacts in the PFM to assemble into the functional pore [Fig. 4(D)]. Therefore, the function of this protease cleaved domain of aerolysin is to mask the interfaces that are involved in oligomerization allowing proaerolysin to maintain a soluble state that is less susceptible to oligomerization. The anthrax toxin protective antigen also uses protease cleavage to remove an N‐terminal 20‐kDa domain (PA_20_) as a prerequisite for oligomerization. The proteolytic site of the cleaved domain contains a furin recognition motif,^9^ suggesting a possible mechanism of activation that involves the spatial localization of PA_83_ with the cell surface of the host. After cleavage, release of the PA_20_ domain exposes a site on PA_63_ to promote oligomerization and to recruit EF and LF toxins.^93^ In both cases described above, the protease cleaved domains are released by host proteases, while monalysin from *Pseudomonas entomophila* can also be activated by AprA, a metallo‐protease co‐secreted by the bacterium.^94^ Structure determination using cryo‐EM combined with complementary biophysical data has helped to predict that the N‐terminal protease cleaved domain of monalysin promotes the interaction of two disk‐shaped nanomers to form a stable doughnut‐like 18‐mer promonalysin with transmembrane segments buried inside the two disks.^95^ The proteolytic cleavage event is proposed to allow the two nanomers to disassociate, exposing the transmembrane segment to undergo the conformational changes necessary to promote pore formation.^95^ Thus, despite the functional variety of the protease cleaved domain, this proteolytic cleavage event appears to be commonly engaged in steps towards pore assembly and maturation as well.

### Target recognition/receptor binding domain

As the name implies, target recognition domains of β‐PFPs coordinate the events that target the protein to specific cell membranes. However, another important function of the target recognition domain common to many PFPs is to facilitate prepore assembly. The targeting of protein monomers to unique entities on the cell surface aids in oligomerization by increasing the local concentration of β‐PFP monomers. These domains sense a diverse set of biological molecules on the target cell surface that includes protein receptors, as well as specific lipids and carbohydrates.

The C‐terminal receptor binding domain of anthrax toxin protective antigen [Fig. 2(A)] recognizes and binds to two cellular receptors: the anthrax toxin receptor (ATR)/tumor endothelial marker 8 (TEM8) and capillary morphogenesis protein 2 (CMG2).^65^, ^96^ Crystallographic structures unveil a detailed interaction between the PA receptor binding domain and the CMG2 receptor.^67^, ^78^ These data indicate that binding is partially attained through interactions involving a metal‐ion‐dependent adhesion site (MIDAS) motif that resides within a von Willebrand factor A (VWA) domain of the CMG2 receptor.^67^, ^78^ Additional interactions formed between the PA PFM and CMG2 aid in distinguishing the targeted receptors from the integrins, which possess motifs that are homologous to the VWA domains of TEM8 and CMG2 receptors.^67^

In addition to specific recognition via protein‐protein interactions, β‐PFPs have adapted domains that can detect the unique composition of the target lipid membrane. The C‐terminal receptor binding domain of lysenin contains a β‐trefoil fold that specifically recognizes the phosphocholine (PC) head‐group and an acyl tail of sphingomyelin (SM).^61^ The crystal structures show that lysenin can adopt an open or a closed state and that interactions with PC are only accessible in the open conformation.^61^ Specifically, lysenin contributes a hydrogen bonding network and a salt bridge that is accessible to PC interactions in the open state, but this interface becomes occluded in the closed state of lysenin. The cryo‐EM structure revealed that in the lysenin pore state, the positioning of this binding pocket is ideally situated for interactions with PC or SM in the membrane [Fig. 2(B)].^28^ Both intrachain and interchain contacts are primarily conserved throughout the receptor‐binding domain of the lysenin nonamer in both the prepore and the pore states [Fig. 4(A,B)]. This observation indicates that the receptor‐binding domain maintains the recognition of targets on cell membranes to stabilize the oligomeric states of lysenin prepore and pore structures [Fig. 4(B)]. Other examples of lipid sensing interactions of β‐PFPs include PlyA, the receptor binding domain of Pleurotolysin, that preferentially recognizes a mixture of SM and cholesterol^97^, ^98^ and a higher affinity of the C‐terminal domain of perfringolysin O for lateral membrane microdomains (rafts) formed by cholesterol.^99^

The recognition of carbohydrate structures on the cell surface is exemplified by aerolysin. The receptor binding domain of aerolysin is comprised of two separate segments [Fig. 2(A)] folding into an N‐terminal small lobe domain and a C‐terminal large lobe domain.^92^ The small lobe domain adopts the lectin fold that recognizes carbohydrates. While the atomic details of the receptor binding domain and receptor interactions are lacking, interactions between aerolysin and glycosylphosphatidylinositol (GPI)‐anchor proteins at residue level have been thoroughly characterized using surface plasmon resonance.^100^ The molecular recognition is modulated through the glycan cores. Only specific types of glycan cores from mammals can bind to aerolysin, but not with GPI‐anchors from other species, such as those in Trypanosomes.^101^ Interestingly, the combined cryo‐EM/X‐ray structure of monalysin, another member of the aerolysin‐like PFPs, reveals that it is devoid of a receptor binding domain.^95^ As such, an articulated mechanism for specific membrane targeting of monalysin requires further investigation.

## Perspectives on Structure‐Guided Elucidation of Pore Assembly and Maturation Mechanisms

Structural information on PFMs of β‐PFPs and particularly the highly conserved features of the intersubunit β‐barrels that comprise the membrane‐spanning pores have enabled researchers to computationally develop models that fit reliably into cryo‐EM density maps even at moderate resolution.^26^, ^102^ Structural studies have uncovered a wealth of information and have provided significant insights towards understanding the mechanisms of activity of β‐PFPs (Supporting Information Table S1). Crystallographic studies have unveiled atomic details and folds of individual domains for β‐PFPs, mainly for the monomeric protein and the oligomeric prepore state, and have been instrumental in defining the molecular basis of specific target recognition. In conjunction with early cryo‐EM studies determined at moderate resolution, these structural studies have helped to elucidate the organization of β‐PFP oligomers in both the prepore and the pore states. More recently, advances in cryo‐EM have enabled researchers to obtain near atomic information that is suitable for building several β‐PFP models *ab initio.* 27, 28, 29 Moreover, through three‐dimensional classification techniques,^103^ cryo‐EM provides the unique opportunity of presenting multiple conformations of the target complex, thereby unleashing the promise of capturing intermediate states during the prepore‐to‐pore transition using the state‐of‐art, time‐resolved cryo‐EM.^104^ Depicting these dynamic and intermediate transitions at atomic detail will undoubtedly facilitate the identification of novel drug targets^105^ that are currently not achievable using more traditional structural biology techniques.

## Supporting information

Supporting Information
