# Supplementary material for: Advances in structure determination by cryo‐EM to unravel membrane‐spanning pore formation
Source: Protein Sci. 2018 Oct 18;27(9):1544–56. doi: 10.1002/pro.3454 (PMC6194281; doi:10.1002/pro.3454)
Supplement: Supplementary file 1 — Supporting Information [file PRO-27-1544-s001.docx]

**Table S1** List of β-PFPs from recent cryo-EM studies revealing the architecture of prepore/pore assemblies.

| Name | TCDB ID | | Species | | Oligomer state | Crystal structures | | Cryo-EM maps | | | | |
| --- | --- | --- | --- | --- | --- | --- | --- | --- | --- | --- | --- | --- |
|  |  |  |  |  |  | PDB | Note | EMDB | Resolution | | Note | |
| Pleurotolysin |  | | *Pleurotus ostreatus* | | 13 | 4OEB ^25^ | pLysA monomer | EMD-2793 ^25^ | 11.0 Å | | pore (4V2T) | |
|  |  | |  |  |  | 4OEJ ^25^ | pLysB monomer | EMD-2794 ^25^ | 15.0 Å | | prepore (4V3A) | |
|  |  | |  |  |  | 4OV8 ^25^ | pLysB TMH1-lock mutant | EMD-2795 ^25^ | 17.0 Å | | prepore (4V3M) | |
|  | 1.C.97.1.1 | |  |  |  | 4V3A ^25^ | pLysB TMH1-lock mutant S-S link | EMD-2796 ^25^ | 14.0 Å | | prepore (4V3N) | |
|  |  |  | |  | | 4V3M ^25^ | pLysB TMH2 helix-lock mutant |  | | | |  |
|  |  |  |  |  |  | 4V3N ^25^ | pLysB TMH2 strand lock mutant S-S link |  |  |  |  |  |
| Lysenin | 1.C.43.1.1 | *Eisenia foetida* | | 9 | | 3ZXG ^60^ | prepore | EMD-8015 ^27^ | 3.1 Å | pore (5GAQ) | |  |
|  |  |  |  |  |  | 5EC5 ^61^ | pore |  | | | |  |
| Anthrax toxin  protective antigen | 1.C.42.1.1 | *Bacillus anthracis* | | 7 | | 1TZO ^95^ | prepore | EMD-6224 ^26^ | 2.9 Å | pore (3J9C) | |  |
| Aerolysin |  | *Aeromonas hydrophila* | | 7 | | 3C0M ^80^ | monomer (Y221G mutant) | EMD-8188 ^28^ | 4.46 Å | post-prepore/quasipore  (5JZW) | |  |
|  | 1.C.4.1.1 |  |  |  |  | 1PRE ^105^ | monomer | EMD-8185 ^28^ | 3.9 Å | prepore (Y221G mutant,  5JZH) | |  |
|  |  |  |  |  |  |  | | EMD-8187 ^28^ | 7.4 Å | pore in LMNG micelle  (K246C/E258C mutant, 5JZT) | |  |
| Perfringolysin O |  | *Clostridium perfringens* | | 30-50 | | 1PFO ^51^ | monomer (F114L mutant) | EMD-1107 ^23^ | 29.0 Å | pore (2BK1) | |  |
|  | 1.C.12.1.1 |  |  |  |  |  | | EMD-1108 ^23^ | 28.0 Å | pore (2BK1) | |  |
|  |  |  |  |  |  |  |  | EMD-1106 ^23^ | 28.0 Å | prepore (2BK2) | |  |
| Monalysin | 1.C.59.3.1 | *Pseudomonas entomophila* | | 9 | | 4MJT ^92^ | prepore | EMD-2698 ^92^ | 17.0 Å |  | |  |
| Suilysin (hemolysin) | 1.C.12.1.8 | *Streptococcus suis* | | 37 | |  |  | EMD-2979 ^24^ | 15.0 Å | prepore | |  |
|  |  |  | |  | |  |  | EMD-2983 ^24^ | 15.0 Å | pore | |  |


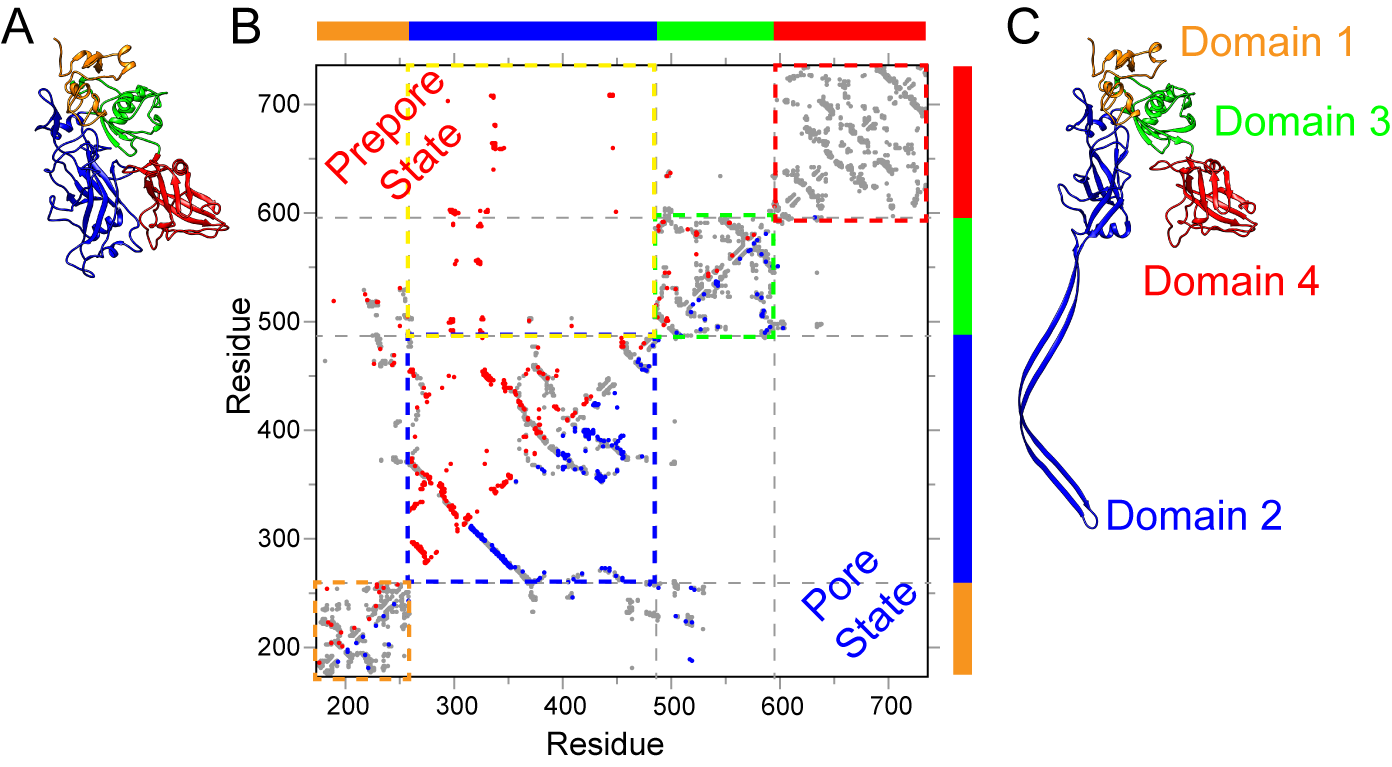


**Figure S1**. Contact map analysis for intra-chain contacts between the prepore state and the pore state of PA. (A) Structure of PA in the prepore state shown in ribbon diagram. Each of the four domains (1-4) are colored in orange, blue, green and red respectively. (B) Contact map of intra-chain contacts as observed in the prepore (upper left triangle area) and in the pore (lower right triangle area) states. Rectangle dash boxes with the same color as each domain are inter-domain contacts, and the yellow rectangle box highlights interactions between domain 2 and domains 3 and 4. (C) Structure of PA in the pore state shown in ribbon diagram.
